# Effects of glucagon-like peptide-1 receptor agonists on alcohol consumption: a systematic review and meta-analysis

**DOI:** 10.1016/j.eclinm.2025.103645

**Published:** 2025-11-14

**Authors:** Reza Eshraghi, Delaram J. Ghadimi, Sara Montazerinamin, Ashkan Bahrami, Yash Kachela, Mahsa Rezasoltani, Mohammad Javad Namazi, Mohsan Subhani, Pouya Ebrahimi, Kaveh Hosseini

**Affiliations:** a Interventional Cardiology Research Center, Isfahan Cardiovascular Research Institute, Isfahan University of Medical Sciences (MUI), Iran; b School of Medicine, Shahid Beheshti University of Medical Sciences, Tehran, Iran; c Rajaei Cardiovascular Medical and Research Centre, Iran University of Medical Sciences, Tehran, Iran; d Student Research Committee, Kashan University of Medical Sciences, Isfahan, Iran; e Aston Medical School, Aston University, Birmingham, B4 7ET, UK; f Department of Haematology, Mayo Clinic, Rochester, MN, USA; g Department of Radiation Oncology, Mayo Clinic, Rochester, MN, USA; h Nottingham Digestive Diseases Centre (NDDC), Translational Medical Sciences, School of Medicine, University of Nottingham, NG7 2UH, UK; i Department of Cardiology, University Hospitals Birmingham, Birmingham, UK; j Cardiovascular Diseases Research Institute, Tehran University of Medical Sciences, Tehran, Iran; k Department of Cardiology, Copenhagen University Hospital - Herlev and Gentofte, Copenhagen, Denmark; l Center for Translational Cardiology and Pragmatic Randomized Trials, Department of Biomedical Sciences, Faculty of Health and Medical Sciences, University of Copenhagen, Denmark

**Keywords:** Alcohol use disorder, GLP-1 receptor agonists, Semaglutide, Neuroimaging, Pharmacotherapy

## Abstract

**Background:**

Alcohol use disorder (AUD) is a major global health burden with high relapse rates and limited treatment uptake. Glucagon-like peptide-1 receptor agonists (GLP-1 RAs), developed for type 2 diabetes and obesity, may also reduce alcohol consumption by modulating central reward pathways.

**Methods:**

We performed a systematic review and meta-analysis following PRISMA guidelines (PROSPERO CRD420251009075). Randomized controlled trials (RCTs) and observational studies were included if they assessed the effect of GLP-1 RAs on alcohol-related outcomes in adults with hazardous drinking or AUD. The search covered all databases from inception to June 1, 2025. The primary outcome was change in Alcohol Use Disorders Identification Test (AUDIT) scores (range 0–40), a validated screening tool assessing hazardous use, dependence, and alcohol-related harm. Alcohol use was defined as self-reported consumption (drinks or units per week, grams per day), with thresholds for risky drinking determined by study-specific definitions (e.g., ≥14 drinks/week for men, ≥7 for women). Secondary outcomes included relapses, abstinence, alcohol-related diagnoses, intoxication or hospitalization, biomarkers (e.g., phosphatidylethanol [PEth], γ-GT), and neuroimaging measures of craving, reward, and cue-reactivity.

**Findings:**

Fourteen studies (four RCTs, ten observational; n = 5,262,268) were included. GLP-1 RAs studied were Semaglutide, liraglutide, dulaglutide, exenatide, and tripeptide. Pooled analysis demonstrated a significant reduction in AUDIT scores (mean difference −7.81 points; 95% CI −9.02 to −6.60; I^2^ = 87.5%). RCTs also reported reduced drinking days, units per drinking day, and cravings particularly with Semaglutide. GLP-1 RA use was associated with reduced alcohol intake, relapse rates, and incidence of alcohol-related diagnoses, especially in individuals with type 2 diabetes or obesity prescribed Semaglutide or liraglutide. Population-based studies showed lower risks of incident and recurrent AUD, intoxication, and hospitalization. Biomarker analyses (PEth, γ-GT) demonstrated reductions with GLP-1 RA use, and neuroimaging studies reported attenuated alcohol cue reactivity and dopaminergic signaling. Weight loss and metabolic improvements reinforced abstinence and treatment adherence.

**Interpretation:**

GLP-1 RAs, particularly Semaglutide and liraglutide, reduce alcohol use as measured by AUDIT scores and show beneficial effects on consumption, relapse, and alcohol-related morbidity. Mechanistic evidence supports modulation of craving and reward pathways. These findings suggest that GLP-1 RAs are promising candidates for repurposing in AUD management, especially for patients with comorbid diabetes or obesity. Large, dedicated RCTs are needed to confirm efficacy and define optimal therapeutic strategies.

**Funding:**

No funding was received for this study.

**Research in context**

**Evidence before this study**

We searched PubMed, EMBASE, Cochrane, Scopus, and ClinicalTrials.gov from inception to June 1, 2025, without language restrictions. Studies were eligible if they reported on the effects of GLP-1 receptor agonists on alcohol consumption, relapse, or alcohol-related diagnoses in adult populations. Risk of bias was assessed using ROBINS-I and RoB 2.0. Prior studies suggested potential efficacy but lacked consistency in population, outcome, and study design.

**Added value of this study**

This is the most comprehensive synthesis to date evaluating GLP-1 receptor agonists across diverse populations and outcomes. It combines randomized clinical trial evidence, large-scale real-world registry data, and mechanistic neurobehavioral insights. Our findings consistently highlight semaglutide and liraglutide as the most effective agents, showing meaningful reductions in alcohol consumption, relapse rates, and alcohol-related diagnoses among individuals with alcohol use disorder (AUD) or hazardous/excessive alcohol use.

**Implications of all the available evidence**

GLP-1 RAs may provide safe dual-action treatment for individuals with AUD and metabolic comorbidities. Future research should focus on targeted RCTs with standardized alcohol-specific endpoints, biomarkers, and mechanistic correlates.

## Introduction

Alcohol use disorder (AUD) is a major public health challenge globally, contributing to over 60 acute and chronic medical conditions and accounting for significant morbidity and mortality.^1^^,^^2^ In the United States, over 25% of adults meet the lifetime diagnostic criteria for AUD, and approximately 11% meet criteria in any given year. Despite its high prevalence, fewer than 10% of affected individuals receive any form of treatment annually, and fewer than 2% receive pharmacotherapy. This reflects a substantial treatment gap.^3^, ^4^, ^5^ Given the limited effectiveness and uptake of current pharmacotherapies for AUD, recent research has explored novel neurobiological targets, particularly those involved in metabolic and appetite-regulating pathways.^6^ Glucagon-like peptide-1 receptor agonists (GLP-1 RAs), which are originally used for type 2 diabetes and obesity management, have shown the potential to be a promising class of medications currently under investigation for alcohol reduction. Glucagon-like peptide-1 receptor agonists (GLP-1 RAs), such as Semaglutide and Exenatide, primarily act through the incretin system to regulate insulin secretion, appetite, and satiety. These agents exert central effects via the gut–brain axis and have been shown to influence reward processing and dopaminergic signaling pathways.^7^ In animal models, GLP-1 receptor activation has been shown to reduce alcohol consumption and alcohol-seeking behavior, likely by modulating mesolimbic dopamine transmission—a key pathway in the neural circuitry of addiction.^8^^,^^9^ Emerging human studies suggest that GLP-1 RAs may attenuate alcohol craving and intake by modulating central reward circuitry.^10^^,^^11^ Functional neuroimaging studies have demonstrated decreased alcohol cue reactivity in brain regions such as the nucleus accumbens, insula, and prefrontal cortex, supporting their role in addiction-related processes.^12^^,^^13^ These effects have reduced alcohol cravings, lower alcohol intake, and decreased relapse rates.^11^ Additionally, functional imaging studies suggest that these medications may alter alcohol cue reactivity in the brain, further contributing to their potential as a treatment for AUD.^14^ Given the growing interest in repurposing GLP-1 RAs for the treatment of AUD, we conducted a systematic review and meta-analysis to evaluate their efficacy and safety in reducing alcohol consumption and related outcomes. This work synthesizes current preclinical and clinical evidence to inform potential therapeutic roles for GLP-1 RAs in AUD management.

## Methods

### Study design and registration

This systematic review and meta-analysis followed the PRISMA 2020 guidelines.^15^ The study protocol was prospectively registered in the PROSPERO database (CRD420251009075).

### Search strategy and selection criteria

We systematically searched PubMed, EMBASE, Scopus, Cochrane Central Register of Controlled Trials, ClinicalTrials.gov, and Google Scholar from database inception through June 1, 2025. The search strategy was developed using the PICO framework and included terms related to “GLP-1 receptor agonists” and “alcohol use.” Full strategies are provided in Supplementary Tables S1 and S2.

Eligible studies included randomized controlled trials (RCTs) and observational studies evaluating the impact of GLP-1 receptor agonists (GLP-1 RAs) on alcohol consumption or alcohol use disorder (AUD) outcomes in adults. Studies were included if they involved individuals ranging from hazardous drinkers to those diagnosed with AUD by validated tools (e.g., AUDIT, DSM-5, or ICD-10).^16^^,^^17^ Primary outcomes were changes in AUDIT scores. Secondary outcomes included alcohol intake levels, relapse rates, healthcare utilization, alcohol-related diagnoses, and neuroimaging endpoints. We excluded non-human, preclinical, review, editorial, or comment articles.

Two reviewers independently screened titles and abstracts and performed full-text review. Discrepancies were resolved through discussion or by a third reviewer. Data extraction was performed in duplicate using a standardized Excel form.

### Ethics

As this study involved no original human or animal data collection, ethical approval was not required. All data were obtained from previously published studies or publicly available databases.

### Study outcomes and data extraction

For each eligible study, we extracted: study design, country, publication year, sample size, characteristics of the study population (including AUD or hazardous drinking definitions), intervention details, and outcome measures. Extracted effect estimates included.

• Mean differences (MD) for AUDIT scores
• Hazard ratios (HR) for relapse and AUD diagnosis
• Odds ratios (OR) for intoxication or healthcare utilization
• Standardized beta coefficients for alcohol consumption change

### Risk of bias assessment

The risk of bias in included RCTs was assessed using the Cochrane RoB 2.0 tool, while non-randomized studies were evaluated using the ROBINS-I tool. One case series was evaluated qualitatively using a narrative risk-of-bias assessment. Domains included confounding, selection bias, classification of interventions, missing data, and outcome measurement.

### Statistical analysis

We conducted meta-analyses using a random-effects model implemented in R (version 4.2.3) with the meta package. The primary pooled effect estimate was the mean difference in AUDIT scores. Between-study heterogeneity was assessed using the I^2^ statistic. We also performed leave-one-out sensitivity analyses to assess robustness of findings and visually inspected funnel plots to assess publication bias.

### Role of the funding source

No funding was received for this study. The authors had full access to all the data in the study and had final responsibility for the decision to submit for publication.

## Results

### Screening and data selection

**Fig. 1:**
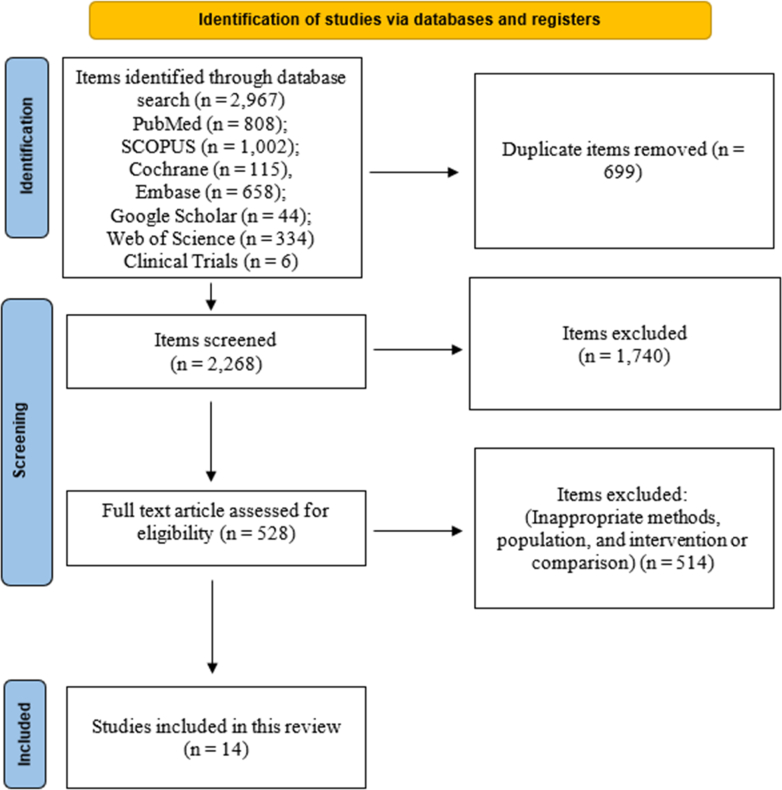
The Prisma flowchart shows the process of study selection.

A literature search was conducted on June 1, 2025, across PubMed, Scopus, Cochrane, and EMBASE. A total of 2967 records were initially identified from multiple electronic databases, including PubMed (n = 808), Embase (n = 658), Scopus (n = 1002), Cochrane (n = 115), Google Scholar (n = 44), Web of Science (n = 334) and ClinicalTrials.gov (n = 6). After removing 699 duplicate records, 2268 unique studies proceeded to the title and abstract screening phase (Fig. 1). A list of excluded full-text articles with reasons is available in Supplementary Tables S3 and S4.

During the screening stage, 1740 studies were excluded based on predetermined eligibility criteria. Subsequently, 528 studies were sought for full-text retrieval; however, 12 studies could not be retrieved. As a result, 516 reports underwent a full-text eligibility assessment. A further 502 studies were excluded at this stage due to methodological inconsistencies, inappropriate study populations, unrelated interventions, or non-relevant comparisons (A list of excluded full-text articles with reasons is available in Supplementary Tables S5. Fourteen studies (three RCTs, a secondary analysis of an RCT, and ten observational studies) met inclusion criteria (Table 1).

**Table 1:** Primary characteristics of the included studies.

First author	Study design	Population	Test group treatment	Control treatment	Test Group (n)	Control group (n)
**O'Farrell, 2025**^18^	Prospective cohort	BMI ≥27 kg/m2 who were drinkers	Liraglutide or Semaglutide	None	231	None
**Quddos, 2023**^19^	Remote Study & Social Media Analysis	Adults self-reporting GLP-1 RAs use via Reddit; off-label for weight loss (BMI ≥30) or T2DM; no prescribed concomitant AUD	Semaglutide or Tirzepatide	Reddit users with no diabetes/obesity meds	104	47
**Richards, 2023**^20^	Case Series	Patients with obesity (BMI: 25.4–47.2 kg/m^2^) treated for weight loss	In-label Semaglutide 0.25–1.00 mg weekly	None (Retrospective Chart Review)	6	None
**Wium-Andersen, 2022**^21^	Nationwide Register-Based Cohort and Self-Controlled Case Series Study	New users of GLP-1 RAs and DPP-4i	GLP-1 RAs	Dipeptidyl Peptidase-4 (DPP-4) inhibitors	38,454	49,222
**Qeadan, 2025**^22^	Retrospective Cohort Study	Adults (≥18 years) with OUD or AUD between 2014 and 2022.	GIP/GLP-1 RAs prescriptions	No GIP/GLP-1 RAs prescriptions	817,309	503,747
**Lähteenvuo, 2025**^23^	Observational Cohort Study	–	Exenatide, Liraglutide, Dulaglutide, and Semaglutide	Nonuse of GLP-1 RAs or AUD medications users	6276	221,590
**Wang, 2024**^24^	Retrospective Cohort Study	Adults with obesity or type 2 diabetes, with or without prior AUD	Semaglutide (Wegovy 2.4 mg for obesity; Ozempic 0.5–1 mg for T2DM)	Other anti-obesity medications	45,797	38,028
**Kuo, 2025**^25^	Retrospective Cohort Study	Adults with type 2 diabetes and AUD (with or without alcohol-related liver disease)	Semaglutide, Liraglutide and Dulaglutide	DPP-4 Inhibitors	6114	5254
**Farokhnia, 2025**^26^	Retrospective Cohort Study	–	Exenatide, Albiglutide, Dulaglutide, Liraglutide, and Semaglutide	DPP-4 Inhibitors and unexposed individuals	30,329	DPP-4 inhibitors: 86,190, eligible unexposed: 3,397,092
**E. Jensen, 2024**^27^	Secondary analysis of a randomized placebo-controlled trial	Adults with alcohol use disorder (AUD) and comorbid obesity (BMI ≥30 kg/m^2^)	Exenatide extended-release (Bydureon®), administered as 2 mg once-weekly injections	Placebo	15	15
**Klausen, 2022**^13^	Randomized, Double-Blined, Placebo-Controlled Clinical Trial	Adults diagnosed with AUD, with ≥5 heavy drinking days in the past 30 days	Exenatide (2 mg subcutaneously once weekly for 26 weeks)	Placebo	62	65
**Probst, 2023**^19^	Randomized, Double-Blinded, Placebo-Controlled Clinical Trial	Adult smokers (ages 18–75) with at least moderate nicotine dependence (Fagerström score ≥5), predominantly obese (BMI >29.9 in ∼91%), undergoing smoking cessation therapy with varenicline and behavioral counseling	Dulaglutide (weekly, subcutaneous 1.5 mg for 12 weeks)	Placebo	76	75
**Hendershot, 2025**^11^	Randomized Clinical Trial	Non–treatment-seeking adults aged 21–65 with AUD, consuming above-threshold levels of alcohol (women >7 drinks/week; men >14) and not attempting to reduce drinking.	Once-weekly subcutaneous Semaglutide (0.25 mg/week for 4 weeks, 0.5 mg/week for 4 weeks, 1.0 mg for 1 week)	Placebo	24	24
V. John, 2025^28^	Observational cohort study (propensity score-matched)	U.S. Veterans with harmful alcohol use (positive AUDIT-C score)	GLP-1 RAs (Semaglutide, Liraglutide, Exenatide, or Dulaglutide)	No GLP-1 RA	8040	8040

### Risk of bias assessment

**Fig. 2:**
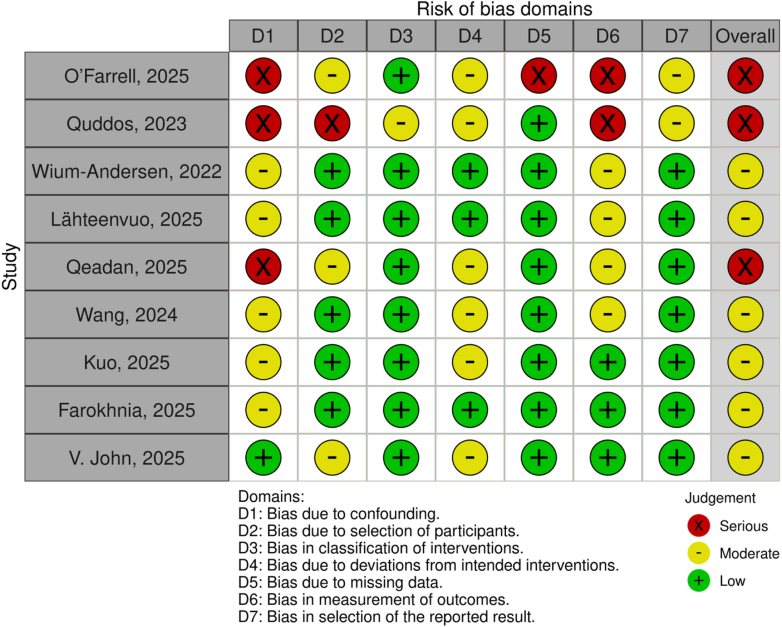
Risk of bias assessment based on the ROBINS-I cochrane handbook guideline.

This ROBINS-I evaluation identifies moderate to serious risk of bias in most non-randomized studies assessing GLP-1 RAs and alcohol use outcomes. Bias due to confounding was common, especially in uncontrolled or self-reported studies. Studies with large sample sizes, registry-based designs, or active comparator groups generally achieved better ratings. Outcome measurement bias was also a concern where alcohol use was assessed solely via self-report. However, most studies had robust intervention classification and low levels of bias due to missing data or selective reporting. These findings underscore the need for rigorous randomized trials and biomarker-based endpoints in future research (Fig. 2).

**Fig. 3:**
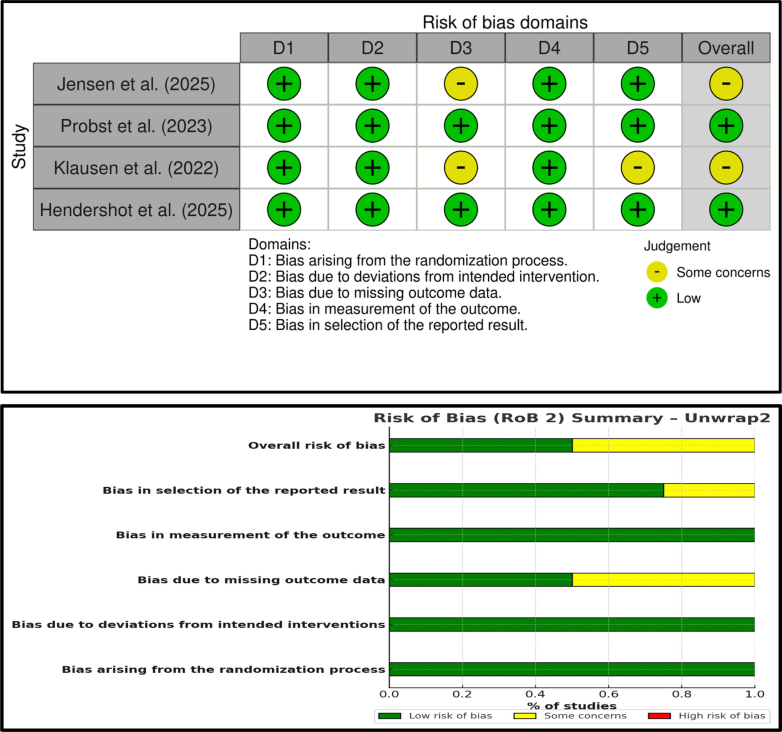
Risk of bias assessment of randomized controlled trials using the RoB 2.0 tool.

The risk of bias assessment using the ROB 2.0 tool across the four included randomized controlled trials revealed that two studies—Probst et al. (2023)^29^ and Hendershot et al. (2025)^11^- were judged to have a low overall risk of bias, with all individual domains rated as low risk. Jensen et al. (2025)^27^ and Klausen et al. (2022)^13^ were judged to have some concerns overall, primarily due to issues in the “bias due to missing outcome data” domain in both studies. Additionally, Klausen et al.^13^ raised some concerns related to deviations from the intended interventions, which further contributed to its overall rating. Although several RCTs demonstrated low risk of bias, most observational studies were rated as having moderate-to-serious risk, primarily due to self-reported outcomes and confounding. Furthermore, many effect estimates were derived from uncontrolled or retrospective datasets, which reduces confidence in causal inference (Fig. 3).

A total of 5,246,188 participants (356 from RCTs, 5,261,912 from observational studies) were included. The pooled mean age was 50.54 years (SD = 10.50), and 56.88% were male. The four RCT studies included 177 participants in the test groups and 179 participants in the control groups. The test treatments consisted of GLP-1 RAs, including Semaglutide (once-weekly doses), Exenatide (2 mg weekly), and Dulaglutide (1.5 mg weekly).

Most participants came from a retrospective observational cohort study. The largest study of GLP-1 RAs recipients was conducted by Qeadan 2025,^22^ including 817,309 patients (almost 78% of the total 1,050,620 recipients). In comparison, 1,040,937 participants were included in the control groups, of which 140,666 were treated with dipeptidyl peptidase 4 (DPP4) inhibitors. The self-reported alcohol measures are detailed in Table 2. The four RCTs included 177 participants treated with GLP-1 RAs (Semaglutide, Exenatide, or Dulaglutide) and 179 participants who received a placebo as the comparator arm.

**Table 2:** Self-report changes in included Studies.

Study	Outcomes
Andersen, 2022^21^	Regarding the alcohol-related events, GLP-1 RAs were compared with the use of DPP-4 and showed ITT-adjusted HR–0–90 days: 0.46 (SD = 0.24–0.86), 90–365 days: 0.98 (SD = 0.64–1.49), 0–365 days: 0.76 (SD = 0.53–1.07), 356–4619 days: 0.72 (SD = 0.60–0.86).
E. Jensen, 2024^27^	Self-reported changes in alcohol consumption were assessed using the Timeline Follow back Method over the past 30 days. At baseline, the Exenatide group had significantly higher AUDIT scores compared to the placebo group. By the end of the study, both groups showed reductions in heavy drinking days and total alcohol consumption, but the differences between the two groups were not statistically significant. Despite the lack of statistically significant differences, both groups exhibited notable reductions in alcohol consumption, with the exenatide group starting with higher levels at baseline.
Farokhnia, 2025^26^	The results showed that GLP-1 RAs recipients reported a significant reduction in their AUDIT-C scores. Specifically, compared to unexposed individuals, the difference-in-difference (DiD) estimate was 0.09 points (95% CI: 0.03, 0.14; p = 0.0025), and compared to DPP-4I recipients, the DiD was 0.11 points (95% CI: 0.05, 0.17; p = 0.0002). These reductions were more pronounced among individuals with baseline AUD or hazardous drinking (AUDIT-C ≥ 8). In contrast, DPP-4I recipients showed no significant change in AUDIT-C scores compared to either the unexposed group or the GLP-1 RAs group, indicating that GLP-1 RAs were effective in reducing alcohol consumption, while DPP-4Is had no effect.
Hendershot, 2025^11^	Once-weekly subcutaneous Semaglutide reduced alcohol consumption during posttreatment laboratory self-administration (β, −0.48; 95% CI of −0.85 and −0.11; P = 0.01) and peak breath alcohol concentration (β, −0.46; 95% CI of −0.87 and −0.06; P = 0.03). Semaglutide substantially reduced drink units per each drinking day (β, −0.41; 95% CI of −0.73 and −0.09; P = 0.04) and weekly alcohol craving (β, −0.39; 95% CI of −0.73 and −0.06; P = 0.01), predicting more significant reductions in heavy drinking over time when compared to placebo (β, 0.84; 95% CI of 0.71 and 0.99; P = 0.04). Semaglutide was also associated with a significant treatment-by-time interaction indicating greater reductions in cigarettes per day in smokers (β, −0.10; 95% CI of −0.16 and −0.03; P = 0.005).
Klausen, 2022^13^	The Exenatide group reduced heavy drinking days by 19.6, compared to 26.8 days in the placebo group, but this difference was not statistically significant (p = 0.37). Similarly, total alcohol intake in 30 days decreased by 1304 g in the Exenatide group and 1313 g in the placebo group, with no significant difference (p = 0.86). Days without alcohol consumption were fewer in the Exenatide group (11.3 days) compared to the placebo group (20.6 days), though this was also not significant (p = 0.11). No significant differences were found in alcohol use disorder scales such as PACS and AUDIT. However, in a subgroup of patients with a BMI greater than 30, Exenatide showed a reduction in both heavy drinking days and total alcohol intake, though these effects were not observed in the overall patient population.
Kuo, 2025^25^	GLP-1 RAs was associated with a lower risk of recurrent AUD (HR: 0.89, 95% CI: 0.81–0.98; p = 0.017)
O'Farrell, 2025^18^	Following the initiation of GLP-1 RAs, from 143 patients with quantifiable intake recorded. 71.8% (n = 188) attended at least two follow-up visits (mean interval: 112 days), and 44.7% (n = 117) had post-intervention alcohol data. No patient reported increased alcohol use. Overall mean alcohol intake decreased significantly from 11.8 ± 1.0 to 4.3 ± 0.5 units/week (p < 0.001). High consumers (≥11 units/week) reduced intake from 23.2 ± 1.8 to 7.8 ± 0.9 units/week, and low consumers (<11 units/week) from 5.5 ± 0.3 to 2.5 ± 0.3 units/week (both p < 0.001). Gender differences in reduction were not statistically significant. A weak positive correlation (r = 0.24, n = 72) was observed between alcohol reduction and weight loss. Notably, even patients who did not reduce alcohol intake (n = 51) experienced weight loss (mean: 7.6 ± 0.6 kg).
Probst, 2023^19^	Relative effect size for a reduction in alcohol intake compared to placebo = 0.71 (95% CI 0.52–0.97, p = 0.04).
Qeadan, 2025^22^	Patients prescribed GIP/GLP-1 RAs exhibited significantly reduced rates of alcohol intoxication compared to those without such prescriptions. Among individuals with AUD, the adjusted incidence rate ratio for alcohol intoxication was 0.50 (95% CI: 0.40–0.63), indicating a statistically significant protective association.
Quddos, 2023^19^	Alterations compared to control: Number of drinking units Semaglutide: B = −1.31 (SE = 0.3, p < 0.001), Tirzepatide = −1.54 (SE = 0.31, p < 0.001). Binge drinking: Semaglutide: B = −2.05 (SE = 0.6, p < 0.001), Tirzepatide: B = −3.8 (SE = 0.68, p < 0.001). AUDIT: Semaglutide: B = −5.1 (SE = 1.3, p < 0.001), Tirzepatide: B = −6.7 (SE = 1.3, p < 0.001).
Richards, 2023^20^	Mean reduction of 9.5 points in AUDIT, p < 0.001; Reduction in alcohol intake = 6.
Wang, 2024^30^	Semaglutide was associated with a significantly lower risk of both incident and recurrent AUD diagnoses compared to non-GLP-1 RAs anti-obesity and anti-diabetic medications, including naltrexone and topiramate. This association was consistent across various subgroups defined by age, gender, race, and the presence or absence of T2DM or obesity. Among patients with no prior history of AUD, Semaglutide users had a notably lower incidence of new AUD diagnoses, while among those with a history of AUD, Semaglutide significantly reduced the risk of recurrence. These protective effects were sustained over 12 months and persisted, though attenuated, over longer follow-up periods.
V. John, 2025^28^	patients treated with GLP-1 RAs had lower odds of having positive AUDIT-C scores during follow-up (adjusted odds ratio (OR) of 0.75; 95% CI 0.68, 0.82; p < 0.0001) compared to controls not receiving GLP-1 RAs therapy. The protective effect was consistent across multiple patient subgroups with or without steatosis or diabetes, notably observed in those without diabetes (adjusted OR of 0.64; 95% CI 0.53, 0.76; p < 0.0001).

#### Reduction in alcohol consumption following GLP-1 RAs use

A consistent finding across diverse studies is the reduction in alcohol consumption, both in quantity and frequency, following the administration of GLP-1 RAs. Kalra (2011) reported that among 42 diabetic patients who consumed alcohol, 78% (33 individuals) reduced their intake after three months of liraglutide treatment, with nine patients ceasing alcohol use entirely and exhibiting significant reductions in MAST scores. Similarly, O'Farrell et al. (2025) documented a decrease in alcohol consumption from 11.8 to 4.3 units per week following GLP-1 RAs initiation.^18^ High consumers (defined as >11 units/week) reduced intake by an average of 15 units, while low consumers demonstrated a 54% reduction (p < 0.001). Richards et al.^20^ found that all six patients treated with Semaglutide showed marked decreases in AUDIT scores, with a mean drop of 9.5 points, transitioning from harmful to low-risk drinking profiles within a short period. In another study, Probst et al. observed a 36% reduction in alcohol consumption among dulaglutide-treated participants compared to placebo (p = 0.004).^29^ Supporting these results, Hendershot et al. (2025) conducted a randomized controlled trial revealing significantly fewer drinking days during weeks 5–8 in participants receiving Semaglutide for moderate AUD (z = −2.93, p = 0.003).^11^ Collectively, these findings highlight a class-wide trend of GLP-1 RAs effectively suppressing alcohol intake across varied populations and methodological approaches.

**Fig. 4:**
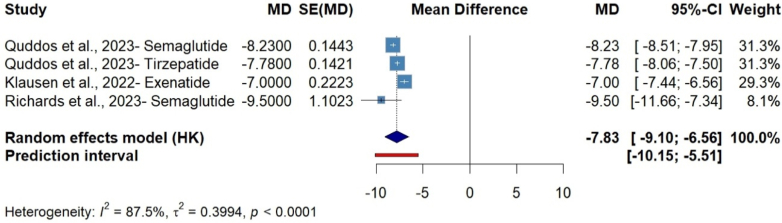
Random-effects meta-analysis showed a significant reduction in symptoms of alcohol use disorder (AUD), as measured by the AUDIT score.

The findings demonstrate a consistent direction of effect across studies, supporting the hypothesis that GLP-1 RAs significantly reduce symptoms of alcohol use disorder (AUD), as measured by the AUDIT score. The random effects model revealed a mean reduction of 7.81 (95% CI: −0.92, −6.60, P = 0.0003, t = −20.53) in AUDIT scores after GLP-1 RAs consumption (Fig. 4). However, significant heterogeneity (I^2^ ≈ 87.5%) was observed, likely attributable to variations in study design (e.g., randomized controlled trials vs. case series), patient populations, and differences in measurement intervals (Supplementary Table S4). To enhance the robustness and interpretability of the results, sensitivity analyses, using the leave-one-out method, were employed. Excluding any studies that did not substantially alter the effect size or significance, underscoring the overall robustness and consistency of the observed effect (Supplementary Figure S1). In addition, funnel plot asymmetry was visually assessed to evaluate publication bias, suggesting potential publication bias (Supplementary Figure S2).

#### Reductions in alcohol-related diagnoses and hospitalizations

Several large-scale population-based investigations have established that GLP-1 RAs are associated with a decreased risk of alcohol-related clinical outcomes, including formal diagnoses and hospitalizations. Wang et al. (2024) demonstrated that Semaglutide use was linked with a 50% reduced risk of recurrent AUD diagnoses (HR = 0.50), a 56% reduced risk of incident AUD among individuals with type 2 diabetes mellitus (HR = 0.56), and a 75% lower risk of recurrent AUD when compared to naltrexone or topiramate (HR = 0.25).^24^ Lähteenvuo et al. (2025) analysed data from 227,868 Swedish patients with AUD.^23^ They found that Semaglutide users had the lowest risk of hospitalization due to AUD (adjusted HR = 0.64) and any substance use disorder (SUD) (aHR = 0.68). Liraglutide also conferred protective effects (AUD aHR = 0.72), while conventional AUD pharmacotherapies, such as disulfiram and acamprosate, did not significantly reduce hospitalization risk (aHR = 0.98). These population-based findings provide compelling real-world evidence that GLP-1 RAs may offer clinically meaningful prevention against serious alcohol-related morbidity.

#### Effectiveness in preventing alcohol intoxication and relapse

GLP-1 RAs use has also been linked to a significantly lower incidence of alcohol intoxication episodes—an important surrogate marker for relapse and acute alcohol-related harm. Qeadan et al. (2025) conducted a comprehensive analysis involving over 817,000 individuals with a history of AUD.^22^ They found that prescriptions for GLP-1/GIP RAs were associated with a 50% reduction in the rate of alcohol intoxication (adjusted incidence rate ratio, aIRR = 0.50). Notably, this protective effect was consistently observed across subgroups with diabetes, obesity, and mental health comorbidities. Additionally, the risk of recurrent intoxication episodes was also significantly lower among GLP-1 RAs users (aHR = 0.47). These findings underscore the potential of GLP-1 RAs to not only reduce alcohol use but also mitigate the risk of relapse and alcohol-related emergencies in diverse patient populations.

#### Neural and behavioral mechanisms: craving, reward, and cue reactivity

Mechanistic studies suggest that GLP-1 RAs exert their alcohol-reducing effects by modulating the brain's reward systems, thereby altering craving, perceived reward, and cue-induced urges. Klausen et al.^13^ demonstrated that exenatide reduced alcohol cue reactivity in both the ventral and dorsal striatum using fMRI imaging in obese individuals with AUD. Furthermore, SPECT imaging revealed that exenatide decreased dopamine transporter availability, indicating attenuated dopaminergic signaling in reward pathways. Complementary to these neuroimaging findings, Quddos et al.^19^ performed a qualitative analysis of over 1500 Reddit posts, with 72% referencing reduced alcohol cravings, aversion, or diminished interest in drinking following GLP-1 RAs use. Hendershot et al. (2025) added to this evidence by showing that Semaglutide diminished the subjective stimulant and sedative effects of alcohol.^11^ Collectively, these studies suggest that GLP-1 RAs disrupt both the neural substrates and subjective reinforcers of alcohol consumption.

#### Weight loss and metabolic improvements may reinforce abstinence

The weight loss and metabolic benefits associated with GLP-1 RAs may synergistically support alcohol reduction by enhancing self-regulation, promoting satiety, and improving psychological well-being. O'Farrell et al. (2025) reported an average weight loss of 7.7 kg over four months in patients treated with GLP-1 RAs, with greater reductions observed among those who also curtailed their alcohol use (correlation coefficient r = 0.24).^18^ Similarly, Hendershot et al. (2025) found a mean body weight reduction of 5% in Semaglutide-treated participants over a nine-week intervention period.^11^ These metabolic improvements may contribute to behavioral reinforcement, fostering sustained abstinence and promoting healthier lifestyle choices in individuals with AUD.

#### Advantages over traditional AUD medications

GLP-1 RAs offer multiple clinical and practical advantages over conventional pharmacotherapies for AUD, which may account for their superior outcomes in recent analyses. Lähteenvuo et al. (2025) found that Semaglutide and Liraglutide significantly reduced hospitalizations related to AUD, whereas standard AUD medications—including disulfiram, acamprosate, and naltrexone—showed no significant benefit (aHR = 0.98).^23^ Additionally, the use of traditional AUD drugs was associated with an elevated risk of suicide (aHR = 1.15), a concerning safety signal not observed with Semaglutide. Wang et al.^24^ further reported that Semaglutide outperformed naltrexone and topiramate in reducing recurrent AUD diagnoses and alcohol intoxication events. These findings support the emerging role of GLP-1 RAs as potentially safer and more efficacious alternatives for the pharmacological management of AUD.

#### Blood phosphatidylcholine (PEth) evaluations

The two studies investigating the effects of exenatide on alcohol consumption in patients with AUD found varying results regarding the alcohol biomarker phosphatidylcholine (PEth). In Klausen et al. study,^13^ although exenatide showed a reduction in PEth levels, this change was not statistically significant (p = 0.64), and there was no significant difference in heavy drinking days or total alcohol intake compared to the placebo group. However, Jensen et al.,^27^ in a secondary analysis focusing on AUD patients with obesity, found that exenatide significantly reduced PEth levels by −0.9 μmol/L at Week 26 (p = 0.03), indicating a delayed but notable effect on alcohol consumption. The reduction in PEth levels was not significant at earlier time points (Weeks 4, 12, and 20), suggesting that the therapeutic effect of exenatide on alcohol consumption may take time to manifest, particularly in individuals with comorbid obesity. While preliminary data from fMRI and PEth support neurobiological plausibility, these findings require validation in adequately powered mechanistic trials.

#### Alcohol-related events

Two studies illustrated that GLP-1 RAs, particularly Semaglutide and Liraglutide, may help reduce alcohol-related events. Andersen et al.^21^ found that GLP-1 RAs were associated with a lower risk of alcohol-related events, especially during the first few months of treatment, compared to DPP-4 inhibitors, with the protective effect being most pronounced initially and diminishing over time. Similarly, Lähteenvuo et al.^23^ reported that the use of Semaglutide and Liraglutide was linked to a reduced risk of hospitalization due to AUD and substance use disorder (SUD), with Semaglutide showing the strongest effect. In this study, besides Semaglutide and Liraglutide, other GLP-1 receptor agonists such as dulaglutide and exenatide were also examined. However, their findings showed that while Dulaglutide and exenatide were associated with a reduced risk of AUD and SUD hospitalizations, their effects were not as pronounced as those of semaglutide and Liraglutide. Specifically, dulaglutide did not show a statistically significant reduction in AUD-related hospitalizations, and Exenatide, though associated with some risk reduction, had less compelling evidence of efficacy compared to semaglutide and Liraglutide.

#### Real-world validation across digital and registry data

The alcohol-reducing effects of GLP-1 RAs have been corroborated through diverse real-world evidence sources, including digital health platforms and international healthcare registries, enhancing their external validity. Quddos et al. employed sentiment analysis and supervised learning techniques on Reddit posts, revealing widespread reports of alcohol aversion and decreased cravings associated with GLP-1 RAs use.^19^ Qeadan et al.^22^ and Wang et al.^24^ utilized large-scale datasets from the U.S. and other countries, yielding consistent results across demographic subgroups, comorbidities, and medication classes. Lähteenvuo et al. (2025) further reinforced these findings in a Nordic cohort, followed by a median of 8.8 years.^23^ Together, these data sources validate the therapeutic potential of GLP-1 RAs in real-world populations affected by AUD.

## Discussion

The real-world applicability of GLP-1 RAs in reducing alcohol consumption is particularly relevant for individuals with comorbid obesity or type 2 diabetes mellitus (T2DM)—populations that often have higher rates of alcohol use and face compounded health risks. Many of the included studies, both randomized and observational, enrolled patients with these metabolic conditions, reflecting a clinical population frequently encountered in practice. The dual benefit of GLP-1 RAs in improving metabolic parameters while simultaneously reducing alcohol intake presents a unique therapeutic opportunity. As such, these agents may be especially well-suited for integrated management strategies targeting both metabolic disease and alcohol use disorder (AUD), offering a pragmatic solution in settings where adherence to traditional AUD treatments is low and comorbidity is high.

Across a diverse set of randomized controlled trials (RCTs) and observational studies, our analysis consistently found that GLP-1 RAs—particularly Semaglutide and Liraglutide—were associated with significant reductions in alcohol intake. These findings echo the direction of earlier preclinical and animal studies, further strengthening the therapeutic rationale.^11^^,^^26^^,^^30^ Nevertheless, important limitations must be acknowledged, including small sample sizes, retrospective study designs, reliance on self-reported outcomes, and variability in AUD diagnostic criteria, clarifying the significant heterogeneity observed in meta-analysis. These factors warrant cautious interpretation of the pooled results.

To contextualize these clinical outcomes, it is important to consider the underlying neurobiological mechanisms that may explain the observed reductions in alcohol use. GLP-1 RAs are believed to modulate central reward pathways, notably dopaminergic transmission within the mesolimbic system—an area critically involved in addiction.^31^, ^32^, ^33^, ^34^ This mechanism is consistent with animal models demonstrating reduced alcohol-seeking behaviors following GLP-1RA administration. These agents target GLP-1 receptors widely distributed across brain regions implicated in reward processing (e.g., nucleus accumbens, ventral tegmental area) as well as stress and cognitive regulation (e.g., hypothalamus, amygdala, prefrontal cortex, and hippocampus).^26^^,^^30^^,^^35^, ^36^, ^37^ Activation of these receptors may dampen the reinforcing effects of alcohol by modulating dopaminergic and GABAergic signaling, thereby reducing both craving and consumption. Furthermore, GLP-1 RAs may attenuate stress-induced drinking by reducing physiological reactivity to withdrawal and anxiety.^38^ Their established effects on appetite and satiety may also contribute to a broader suppression of reward-driven behaviors, including alcohol use. Human neuroimaging studies provide preliminary support for these mechanisms, with reduced alcohol cue reactivity observed in key reward-related brain regions following treatment with agents like exenatide and semaglutide.^13^

The clinical relevance of GLP-1 RAs is further underscored by their association with reductions in alcohol-related hospitalizations and intoxication episodes, providing real-world validation of their therapeutic potential. Among the agents studied, semaglutide consistently demonstrated the most robust effects across both clinical trials and large-scale population cohorts. Specifically, data from Wang et al. and Lähteenvuo et al. indicate that semaglutide significantly lowers the risk of both incident and recurrent AUD diagnoses, especially among individuals with coexisting obesity or type 2 diabetes mellitus—populations known to be at heightened risk for alcohol-related harm. These findings position semaglutide as a compelling pharmacologic candidate for the treatment of AUD.^23^^,^^24^

However, the therapeutic efficacy of GLP-1 RAs was not uniformly observed across all included studies. For example, Klausen et al.,^13^ reported that exenatide did not significantly reduce alcohol consumption or the number of heavy drinking days when compared to placebo, suggesting that treatment response may vary by specific agent, dosing regimen, or patient characteristics. Notably, several studies observed more pronounced benefits among individuals with obesity, implying that metabolic improvements—such as weight loss and enhanced satiety—may contribute synergistically to reductions in alcohol intake.^18^ This is supported by the findings of O'Farrell et al., where alcohol reduction was positively, albeit modestly, correlated with weight loss. These metabolic–behavioral interactions further reinforce the hypothesis that GLP-1 RAs may exert dual benefits by addressing both physical and neurobehavioral components of AUD.

Interestingly, even in studies where behavioral outcomes were inconclusive, neuroimaging data provided additional insight. Exenatide, for example, significantly attenuated cue-induced activation in reward-related brain regions such as the ventral striatum and septal area—areas that are critically involved in addiction pathways. While these mechanistic findings are promising, the overall evidence base remains heterogeneous in terms of study design, sample characteristics, and outcome measures. Importantly, despite the inclusion of over 77,000 individuals in diabetes-related GLP-1 RA trials, few have systematically evaluated alcohol-specific endpoints. This limits the strength and generalizability of the current conclusions and highlights the need for targeted research in this area.

Looking forward, several ongoing clinical trials—summarized in Supplementary Table S6—are poised to clarify the role of GLP-1 RAs in AUD management. These include Phase 2 studies examining semaglutide in individuals with comorbid obesity, fatty liver disease, and high-risk drinking behaviors. Primary endpoints range from reductions in alcohol intake and cravings to biochemical and imaging-based markers of treatment response. These trials, taking place across North America and Europe, are scheduled for completion between 2025 and 2030 and are expected to provide critical data to guide clinical translation.

To aid interpretation, it is important to clearly distinguish between findings derived from RCTs and those from observational studies. RCTs, such as those by Hendershot et al., Probst et al., Klausen et al., and Jensen et al., provide more robust causal evidence, demonstrating that GLP-1 RAs, particularly semaglutide and liraglutide, significantly reduce alcohol consumption compared to placebo. These trials often used standardized designs and controlled settings, enhancing internal validity. In contrast, most observational studies contributed valuable real-world insights but were more susceptible to confounding, selection bias, and variability in outcome measurement. While large-scale cohorts such as those by Wang et al., Qeadan et al., and Lähteenvuo et al. consistently showed reductions in alcohol-related events (e.g., AUD diagnoses, intoxication, hospitalization), these associations should be viewed as hypothesis-generating rather than confirmatory. This distinction is crucial for informing both clinical application and future research, emphasizing the need for additional RCTs to verify the promising trends observed in real-world settings.

While neuroimaging and biomarker studies provide important mechanistic insights, these findings should be interpreted cautiously. Both the functional MRI (fMRI) and SPECT studies included in this review were based on relatively small sample sizes and lacked replication across diverse populations. Similarly, evidence regarding reductions in phosphatidylcholine (PEth)—an objective biomarker of alcohol intake—was limited to a few trials with modest power and variable reporting intervals. As such, these preliminary data support biological plausibility but fall short of establishing definitive causal pathways. Larger, standardized mechanistic studies are needed to validate these early observations and clarify the neurobiological effects of GLP-1 RAs on alcohol use.

This review has potential limitations. First, there was considerable heterogeneity across studies in terms of population characteristics, study designs, GLP-1 RA agents, dosages, and outcome measures, limiting the comparability and strength of pooled results. Second, many studies relied on self-reported alcohol use (e.g., AUDIT, Timeline Follow-back), which are prone to recall and social desirability bias. Although some studies included biomarkers like PEth, these were inconsistently used. Third, much of the evidence came from observational studies with moderate-to-serious risk of bias, particularly due to confounding and reliance on retrospective data without biomarker validation.

Fourth, the neurobiological evidence is limited, with only a few small neuroimaging studies suggesting mechanistic plausibility. These findings require confirmation in larger, dedicated trials. Lastly, the generalizability of results is constrained, as most participants had obesity or diabetes and were from high-income countries. The effectiveness of GLP-1 RAs in broader or underserved AUD populations remains unclear. Additionally, sex- and gender-specific data were inconsistently reported across included studies, limiting the ability to perform subgroup analyses. Future research should ensure disaggregated reporting by sex and gender to assess potential differential effects of GLP-1 RAs.

In summary, GLP-1 receptor agonists—particularly semaglutide and liraglutide—emerge from this review as promising agents for reducing alcohol consumption and related harms. Evidence from both randomized trials and real-world observational studies supports their multifaceted benefits, including improvements in metabolic health, reduction of alcohol-related diagnoses, and attenuation of neurobehavioral drivers of addiction. If confirmed in future rigorously designed studies, GLP-1 RAs may address a major treatment gap in AUD, particularly for patients with coexisting obesity or diabetes, two populations often underserved by conventional AUD therapies.

Nonetheless, these findings should be interpreted with caution until substantiated by additional trials specifically designed to assess alcohol-related outcomes. Larger, longer-term randomized controlled trials with standardized measures of alcohol use, biomarker validation, and neurobiological endpoints are essential to optimize treatment strategies, understand patient subgroups most likely to benefit, and assess the durability of effects over time.

## Contributors

Reza Eshraghi and Pouya Ebrahimi conceived and designed the study. Delaram J Ghadimi and Sara Montazerinamin performed the literature search and study selection. Ashkan Bahrami and Mahsa Rezasoltani extracted the data and assessed study quality. Mohammad Javad Namazi and Mohsan Subhani conducted statistical analyses and interpreted the results. Yash Kachela and Reza Eshraghi drafted the initial manuscript. Kaveh Hosseini and Pouya Ebrahimi supervised the project and led the revisions.

Reza Eshraghi, Sara Montazerinamin, and Pouya Ebrahimi accessed and verified the underlying data. All authors critically reviewed the manuscript for intellectual content, approved the final version, and agreed to be accountable for all aspects of the work.

## Data sharing statement

No datasets were generated or analyzed during the current study. All data used were obtained from previously published studies and are publicly available. Further information about the data sources can be obtained from the corresponding author upon reasonable request.

## Declaration of interests

All authors declare no competing interests.
