# Supplementary material for: Effects of glucagon-like peptide-1 receptor agonists on alcohol consumption: a systematic review and meta-analysis
Source: eClinicalMedicine. 2025 Nov 14;90:103645. doi: 10.1016/j.eclinm.2025.103645 (PMC12663662; doi:10.1016/j.eclinm.2025.103645)
Supplement: Supplementary Figures and Tables [file mmc1.docx]

**Supplementary Table S1:** Search strategy developed using the PICO framework, including primary keywords and Boolean operators for PubMed, EMBASE, Cochrane, and Scopus.

| Component | Details |
| --- | --- |
| Population | Adults with occasional, excessive alcohol use, or diagnosed Alcohol Use Disorder (AUD), including subgroups with comorbid obesity or type 2 diabetes mellitus (T2DM). |
| Intervention | Glucagon-Like Peptide-1 Receptor Agonists (GLP-1 RAs), including Semaglutide, Liraglutide, Dulaglutide, Exenatide, and Tirzepatide. Doses varied by agent and indication (e.g., 2.4 mg weekly for Semaglutide; 2 mg weekly for Exenatide). |
| Comparator | Placebo, no treatment, or active comparators (e.g., DPP-4 inhibitors, other anti-obesity or anti-diabetic medications such as naltrexone or topiramate). |
| Outcomes | **Primary**: Change in alcohol use (e.g., AUDIT score, alcohol units/week, drinking days, relapse).  **Secondary**: Alcohol-related events (e.g., intoxication, hospitalizations, AUD diagnoses), neurobiological markers (e.g., PEth, fMRI cue reactivity), smoking behavior, weight change, craving, and adverse events. |
| Setting | International studies, including real-world data from EHRs, nationwide registries (Denmark, USA, Sweden), academic clinical trials, and social media-derived surveys. |
| Study Designs Included | Randomized controlled trials (n=4), retrospective and prospective observational studies (n=10), including large EHR cohorts and case series. Total participants: >5.2 million. |

**Supplementary Table S2:** Full database-specific search strings for reproducibility across all databases queried through June 1, 2025.

| Database | Search Syntax |
| --- | --- |
| PubMed | ( "GLP-1 receptor agonist" OR "glucagon-like peptide-1 receptor agonist" OR "GLP1-RA" OR "incretin mimetic" OR exenatide OR liraglutide OR semaglutide OR dulaglutide OR albiglutide OR lixisenatide OR efpeglenatide OR tirzepatide OR "GLP-1 analogue" OR "GLP-1 analog" OR "GLP-1 based therapy" OR "Glucagon-Like Peptide 1 Receptor"[MeSH Terms] OR "Glucagon-Like Peptide 1"[MeSH Terms] OR "Incretins"[MeSH Terms] ) AND ( alcohol OR ethanol OR "alcohol consumption" OR "alcohol intake" OR "alcohol use" OR "alcohol use disorder" OR AUD OR "alcohol dependence" OR "alcohol abuse" OR "alcohol-related disorder*" OR "binge drinking" OR "hazardous drinking" OR "heavy drinking" OR "problem drinking" OR "substance use" OR "substance use disorder" OR SUD OR "substance abuse" OR "substance dependence" OR "drug use" OR "drug abuse" OR addiction OR "chemical dependency" OR "reward pathway" OR craving OR reinforcement OR "psychoactive substance*" OR "compulsive use" OR "Alcohol-Related Disorders"[MeSH Terms] OR "Alcohol Drinking"[MeSH Terms] OR "Substance-Related Disorders"[MeSH Terms] OR "Substance Abuse, Oral"[MeSH Terms] OR "Behavior, Addictive"[MeSH Terms] OR "Reward"[MeSH Terms] OR "Craving"[MeSH Terms] ) |

**Supplementary Table S3:** List of excluded full-text studies with specific reasons for exclusion, organized by PRISMA domains.

| **Reason for Exclusion** | **Number of Studies** |
| --- | --- |
| Inappropriate study design (e.g., reviews, editorials, case reports without primary data) | 144 |
| Non-human/preclinical studies | 106 |
| No assessment of GLP-1 RA as intervention | 68 |
| Alcohol outcomes not reported or irrelevant | 67 |
| Duplicate data or overlapping datasets | 31 |
| Population not meeting inclusion criteria (e.g., pediatric, non-AUD) | 46 |
| Non-English and non-translatable articles | 18 |
| Full text not retrievable | 12 (as noted) |
| Insufficient data for analysis | 10 |
| Total excluded studies | 502 |

**Supplementary Table S4:** Characteristics of excluded studies that were borderline eligible but failed to meet one or more inclusion criteria (e.g., outcome reporting, study design).

| Full Study Title | Trial Label | Reason for Exclusion |
| --- | --- | --- |
| Liraglutide reverses pronounced insulin-associated weight gain, improves glycemic control and decreases insulin dose in patients with type 2 diabetes: a 26 week, randomized clinical trial (ELEGANT)^1^ | Effect of Liraglutide on Insulin-Associated Weight Gain | Alcohol only reported in baseline characteristics; no outcome or subgroup analysis on alcohol use. |
| Liraglutide safety and efficacy in patients with non-alcoholic steatohepatitis (LEAN): a multicenter, double-blind, randomized, placebo-controlled phase 2 study^2^ | LEAN study in NASH | Patients with high alcohol consumption excluded; no alcohol-related outcomes evaluated. |
| Neuropsychiatric Safety of Liraglutide 3.0 mg for Weight Management: SCALE Pooled Analysis^3^ | SCALE pooled neuropsychiatric safety analysis | Focused on depression and suicidality; alcohol not assessed. |
| The effect of liraglutide on renal function: A randomized clinical trial^4^ | Liraglutide added to insulin in T1DM | No mention of alcohol use, intake, or outcomes. |
| Efficacy and safety of liraglutide versus placebo added to basal insulin analogues (with or without metformin) in patients with type 2 diabetes: a randomized, placebo-controlled trial^5^ | RCT on Liraglutide with basal insulin | No alcohol-related data or subgroup analysis. |
| HARMONY 3: 104-week randomized, double-blind, placebo- and active-controlled trial assessing the efficacy and safety of albiglutide compared with placebo, sitagliptin, and glimepiride in patients with type 2 diabetes taking metformin^6^ | HARMONY 3 | No alcohol use data reported or analyzed. |
| PIONEER 1: Randomized Clinical Trial of the Efficacy and Safety of Oral Semaglutide Monotherapy in Comparison with Placebo in Patients with Type 2 Diabetes^7^ | PIONEER trial extension | No alcohol-related endpoints or exclusion criteria involving alcohol. |
| Efficacy and Safety of Once-Weekly Efpeglenatide Monotherapy Versus Placebo in Type 2 Diabetes: The AMPLITUDE-M Randomized Controlled Trial^8^ | AMPLITUDE-M | No alcohol intake or behavioral data evaluated. |
| Daily Oral GLP-1 Receptor Agonist Orforglipron for Adults with Obesity^9^ | Orforglipron in obesity | No alcohol-related criteria or outcomes. |
| Oral semaglutide versus subcutaneous liraglutide and placebo in type 2 diabetes (PIONEER 4): a randomised, double-blind, phase 3a trial^10^ | PIONEER-4 Trial | No alcohol-related data collected or analyzed. |
| Efficacy and Safety of Liraglutide Versus Placebo as Add-on to Glucose-Lowering Therapy in Patients with Type 2 Diabetes and Moderate Renal Impairment (LIRA-RENAL): A Randomized Clinical Trial^11^ | Liraglutide in Type 2 Diabetes | No reference to alcohol consumption or outcomes. |
| Efficacy and Safety of Liraglutide vs. Placebo When Added to Basal Insulin Analogues in Subjects with Type 2 Diabetes (LIRA-ADD2BASAL): A Randomized, Placebo-Controlled Trial^12^ | RCT on Liraglutide with Insulin | Alcohol not assessed or reported in any domain. |
| Dulaglutide and cardiovascular outcomes in type 2 diabetes (REWIND): a double-blind, randomised placebo-controlled trial^13^ | REWIND Trial | Alcohol use not recorded or discussed in baseline or outcome data. |
| Liraglutide reverses pronounced insulin-associated weight gain, improves glycaemic control and decreases insulin dose in patients with type 2 diabetes: A 26 week, randomised clinical trial (ELEGANT)^14^ | ELEGANT trial | Alcohol not included as a variable; excluded if abuse suspected. |
| Weight-Related Quality of Life, Health Utility, Psychological Well-Being, and Satisfaction with Exenatide Once Weekly Compared with Sitagliptin or Pioglitazone After 26 Weeks of Treatment^15^ |  | No alcohol data collected or analyzed. |
| A Randomized, Controlled Trial of 3.0 mg of Liraglutide in Weight Management^16^ | SCALE Obesity Diabetes | Alcohol use not reported; excluded from safety endpoints. |
| Efficacy and Safety of Liraglutide 3.0 mg in Individuals with Overweight or Obesity and Type 2 Diabetes Treated with Basal Insulin: The SCALE Insulin Randomized Controlled Trial^17^ | SCALE Insulin | Alcohol excluded in eligibility but not analyzed or reported. |
| Two-year effects of semaglutide in adults with overweight or obesity: the STEP 5 trial^18^ | STEP 5 | No alcohol variables present; not part of study endpoints. |
| Liraglutide Improves Glycemic Control in Type 2 Diabetics Treated with Multiple Daily Insulin Injections^19^ | Effect on Glycemic Control with Basal Insulin | No mention or analysis of alcohol use or related behaviors. |
| Liraglutide, a once-daily human GLP-1 analogue, added to a sulphonylurea over 26 weeks produces greater improvements in glycaemic and weight control compared with adding rosiglitazone or placebo in subjects with Type 2 diabetes (LEAD-1 SU)^20^ | Add-on to Sulfonylurea Monotherapy (LEAD-1 SU) | Alcohol use not reported, analyzed, or excluded. |
| Liraglutide reduces hyperglycaemia and body weight in overweight, dysregulated insulin-pump-treated patients with type 1 diabetes: The Lira Pump trial—a randomized, double-blinded, placebo-controlled trial^21^ | The Lira Pump trial | Alcohol not assessed; excluded patients with substance misuse but did not report data. |
| Efficacy and safety of switching from sitagliptin to liraglutide in subjects with type 2 diabetes (LIRA‐SWITCH): a randomized, double‐blind, double‐dummy, active‐controlled 26‐week trial^22^ | LIRA-SWITCH | No alcohol data collected or mentioned. |
| Durable efficacy of liraglutide in patients with type 2 diabetes and pronounced insulin-associated weight gain: 52-week results from the Effect of Liraglutide on insulin-associated wEight GAiN in patients with Type 2 diabetes' (ELEGANT) randomized controlled trial^23^ | ELEGANT trial | Baseline alcohol use reported descriptively only; not analyzed further. |
| Efficacy and Safety of Liraglutide Versus Placebo as Add-on to Glucose-Lowering Therapy in Patients With Type 2 Diabetes and Moderate Renal Impairment (LIRA-RENAL): A Randomized Clinical Trial^24^ | LIRA-RENAL | No alcohol-related variable reported or controlled for; excluded those with drug/alcohol abuse history. |
| Semaglutide 2·4 mg once a week in adults with overweight or obesity, and type 2 diabetes (STEP 2): a randomised, double-blind, double-dummy, placebo-controlled, phase 3 trial^25^ | STEP 2: Semaglutide 2.4mg in T2DM | No mention or stratification by alcohol use; exclusion not detailed. |
| Liraglutide as add-on to sodium-glucose co-transporter-2 inhibitors in patients with inadequately controlled type 2 diabetes: LIRA-ADD2SGLT2i, a 26-week, randomized, double-blind, placebo-controlled trial^26^ | LIRA-ADD2SGLT2i | No alcohol-related data collected or analyzed; endpoints were strictly glycemic and weight-based |
| Combination therapy with insulin glargine and exenatide: real-world outcomes in patients with type 2 diabetes^27^ | Exenatide + Insulin Glargine | No mention of alcohol use or consumption behaviors in design, analysis, or exclusion criteria |
| Tolerability and efficacy of exenatide and titrated insulin glargine in adult patients with type 2 diabetes previously uncontrolled with metformin or a sulfonylurea: A multinational, randomized, open-label, two-period, crossover noninferiority trial^28^ | Exenatide + Glargine in T2D | Alcohol not recorded; study focused on glycemic control, weight, insulin, and GI adverse effects. |
| Efficacy and safety of autoinjected exenatide once‐weekly suspension versus sitagliptin or placebo with metformin in patients with type 2 diabetes: The DURATION‐NEO‐2 randomized clinical study^28^ | DURATION‐NEO‐2 | Focused on QOL, satisfaction, psychological effects—no alcohol variables or discussion of alcohol use behavior. |
| PIONEER 1: Randomized Clinical Trial of the Efficacy and Safety of Oral Semaglutide Monotherapy in Comparison with Placebo in Patients with Type 2 Diabetes^29^ | PIONEER 1 | No alcohol-related data reported or analyzed; trial focused on glycemic and weight endpoints only. |
| Efficacy and safety of once-weekly efpeglenatide in people with suboptimally controlled type 2 diabetes: The AMPLITUDE-D, AMPLITUDE-L and AMPLITUDE-S randomized controlled trials^30^ | AMPLITUDE-D, -L, -S | No assessment or analysis of alcohol intake; endpoints focused on glycemic control, body weight, and safety. |

**Supplementary Table S5:** Summary of ongoing or planned clinical trials evaluating GLP-1 receptor agonists for alcohol use reduction, including trial registry IDs, endpoints, and estimated completion dates.

| **Trial ID** | **Title** | **Intervention** | **Inclusion Criteria** | **Sample Size** | **Phase** | **Status** | **Primary Outcome Measure** | **Start- Completion Date** | **Location** |
| --- | --- | --- | --- | --- | --- | --- | --- | --- | --- |
| [NCT05895643](https://clinicaltrials.gov/study/NCT05895643) | Semaglutide for Alcohol Use Disorder and Comorbid Obesity | Semaglutide (2.4 mg/week) vs. Placebo | Diagnosed with alcohol use disorder and comorbid obesity (BMI ≥30 kg/m2), aged 18-70, heavy drinking defined as >6 days with alcohol consumption over 4 units for women or 5 units for men | 108 | Phase 2 | Recruiting | Change in heavy drinking days (TLFB) over 26 weeks | June 2023, Sept 2025 | USA (Yale University) |
| NCT06546384 | Semaglutide for Alcohol Consumption, Metabolism, and Liver Parameters in Obesity | Semaglutide (titrated up to 2.4 mg/week) vs. Control (counselling) | BMI ≥28 or ≥35, diagnosed with fatty liver disease, and alcohol use disorder (AUDIT-C ≥4 for women, ≥5 for men), aged 18-80, with no contraindications for semaglutide or study interventions | 64 | NA | Recruiting | Proportion of patients achieving total alcohol abstinence (measured by negative PEth test) | June 2025, April 2027 | Switzerland (University Hospital Bern) |
| [NCT06015893](https://clinicaltrials.gov/study/NCT06015893) | Semaglutide Therapy for Alcohol Reduction (STAR) | Semaglutide (2.4 mg/week) vs. Placebo | Adults (18+) with AUD; consuming >7 drinks/week (women) or >14 drinks/week (men); able to participate in behavioral therapy sessions. | 52 | Phase 2 | Recruiting | Difference in weekly alcohol consumption; safety and tolerability (adverse events). | Oct 2023, Dec 2030 | USA (NIDA, Baltimore) |
| NCT05892432 | Semaglutide (Rybelsus) for Alcohol Use Disorder | Semaglutide (oral, titrated 3 mg to 7 mg/day) vs. Placebo | Adults (≥21 years) with moderate/severe AUD, BMI ≥25, seeking AUD treatment | 135 | Phase 2 | Recruiting | Change in alcohol cue-elicited craving scores from baseline to week 6 | Jan 2024, June 2025 | USA (University of Colorado) |
| [NCT05891587](https://clinicaltrials.gov/study/NCT05891587) | Semaglutide Therapy for Alcohol Reduction - Tulsa | Semaglutide (0.25–1.0 mg/week) vs. Placebo | Adults (≥18 years) with AUD; consuming >7 drinks/week (women) or >14 drinks/week (men); BMI ≥25 kg/m² | 80 | Phase 2 | Recruiting | Change in weekly number of alcoholic drinks (Drinks Per Week) from baseline to Week 13 | July 2023, Dec 2025 | USA (Tulsa, Oklahoma) |
| [NCT06409130](https://clinicaltrials.gov/study/NCT06409130) | Effects of NNC0194-0499, Semaglutide, and Cagrilintide on Alcohol-related Liver Disease | NNC0194-0499, Semaglutide, Cagrilintide vs. Placebo | Adults (≥18 years) with alcohol-related liver disease; ELF ≥9.0 units; ≥5 years heavy alcohol use | 240 | Phase 2 | Recruiting | Change in Enhanced Liver Fibrosis (ELF) score at week 28 | May 2024, Jan 2026 | Multiple international sites |

**
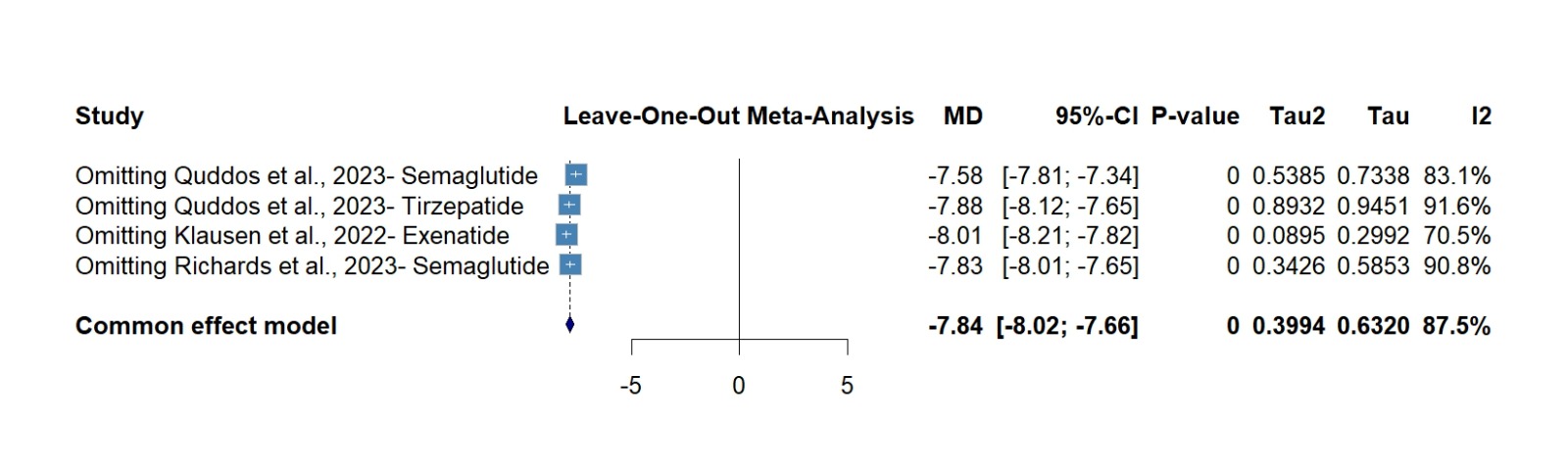
Supplementary Figure 1:** Leave-One-Out sensitivity analysis demonstrating the influence of individual studies on the pooled effect size of AUDIT score reduction.


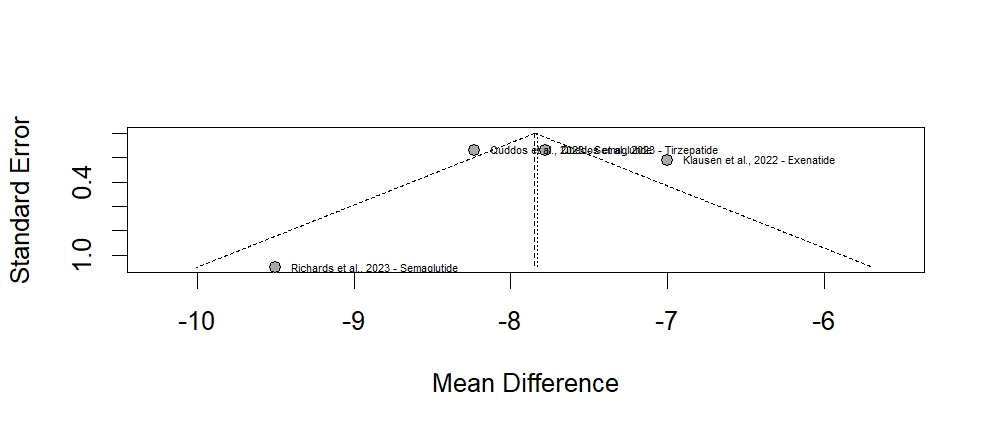


**Supplementary Figure 2:** Funnel plot assessing the risk of publication bias across studies reporting on AUDIT score changes following GLP-1 RA use.

**References**

1 de Wit HM, Vervoort GMM, Jansen HJ, de Grauw WJC, de Galan BE, Tack CJ. Liraglutide reverses pronounced insulin-associated weight gain, improves glycaemic control and decreases insulin dose in patients with type 2 diabetes: a 26 week, randomised clinical trial (ELEGANT). *Diabetologia* 2014; 57: 1812–9.

2 Armstrong MJ, Gaunt P, Aithal GP, *et al.* Liraglutide safety and efficacy in patients with non-alcoholic steatohepatitis (LEAN): a multicentre, double-blind, randomised, placebo-controlled phase 2 study. *The Lancet* 2016; 387: 679–90.

3 O’Neil PM, Aroda VR, Astrup A, *et al.* Neuropsychiatric safety with liraglutide 3.0 mg for weight management: Results from randomized controlled phase 2 and 3a trials. *Diabetes Obes Metab* 2017; 19: 1529–36.

4 von Scholten BJ, Persson F, Rosenlund S, *et al.* The effect of liraglutide on renal function: A randomized clinical trial. *Diabetes Obes Metab* 2017; 19: 239–47.

5 Ahmann A, Rodbard HW, Rosenstock J, *et al.* Efficacy and safety of liraglutide versus placebo added to basal insulin analogues (with or without metformin) in patients with type 2 diabetes: a randomized, placebo‐controlled trial. *Diabetes Obes Metab* 2015; 17: 1056–64.

6 Ahrén B, Johnson SL, Stewart M, *et al.* HARMONY 3: 104-Week Randomized, Double-Blind, Placebo- and Active-Controlled Trial Assessing the Efficacy and Safety of Albiglutide Compared With Placebo, Sitagliptin, and Glimepiride in Patients With Type 2 Diabetes Taking Metformin. *Diabetes Care* 2014; 37: 2141–8.

7 Aroda VR, Rosenstock J, Terauchi Y, *et al.* PIONEER 1: Randomized Clinical Trial of the Efficacy and Safety of Oral Semaglutide Monotherapy in Comparison With Placebo in Patients With Type 2 Diabetes. *Diabetes Care* 2019; 42: 1724–32.

8 Frias JP, Choi J, Rosenstock J, *et al.* Efficacy and Safety of Once-Weekly Efpeglenatide Monotherapy Versus Placebo in Type 2 Diabetes: The AMPLITUDE-M Randomized Controlled Trial. *Diabetes Care* 2022; 45: 1592–600.

9 Wharton S, Blevins T, Connery L, *et al.* Daily Oral GLP-1 Receptor Agonist Orforglipron for Adults with Obesity. *New England Journal of Medicine* 2023; 389: 877–88.

10 Pratley R, Amod A, Hoff ST, *et al.* Oral semaglutide versus subcutaneous liraglutide and placebo in type 2 diabetes (PIONEER 4): a randomised, double-blind, phase 3a trial. *The Lancet* 2019; 394: 39–50.

11 Davies MJ, Bain SC, Atkin SL, *et al.* Efficacy and Safety of Liraglutide Versus Placebo as Add-on to Glucose-Lowering Therapy in Patients With Type 2 Diabetes and Moderate Renal Impairment (LIRA-RENAL): A Randomized Clinical Trial. *Diabetes Care* 2016; 39: 222–30.

12 Ahmann A, Rodbard HW, Rosenstock J, *et al.* Efficacy and safety of liraglutide versus placebo added to basal insulin analogues (with or without metformin) in patients with type 2 diabetes: a randomized, placebo‐controlled trial. *Diabetes Obes Metab* 2015; 17: 1056–64.

13 Gerstein HC, Colhoun HM, Dagenais GR, *et al.* Dulaglutide and cardiovascular outcomes in type 2 diabetes (REWIND): a double-blind, randomised placebo-controlled trial. *The Lancet* 2019; 394: 121–30.

14 de Wit HM, Vervoort GMM, Jansen HJ, de Grauw WJC, de Galan BE, Tack CJ. Liraglutide reverses pronounced insulin-associated weight gain, improves glycaemic control and decreases insulin dose in patients with type 2 diabetes: a 26 week, randomised clinical trial (ELEGANT). *Diabetologia* 2014; 57: 1812–9.

15 Best JH, Rubin RR, Peyrot M, *et al.* Weight-Related Quality of Life, Health Utility, Psychological Well-Being, and Satisfaction With Exenatide Once Weekly Compared With Sitagliptin or Pioglitazone After 26 Weeks of Treatment. *Diabetes Care* 2011; 34: 314–9.

16 Pi-Sunyer X, Astrup A, Fujioka K, *et al.* A Randomized, Controlled Trial of 3.0 mg of Liraglutide in Weight Management. *New England Journal of Medicine* 2015; 373: 11–22.

17 Garvey WT, Birkenfeld AL, Dicker D, *et al.* Efficacy and Safety of Liraglutide 3.0 mg in Individuals With Overweight or Obesity and Type 2 Diabetes Treated With Basal Insulin: The SCALE Insulin Randomized Controlled Trial. *Diabetes Care* 2020; 43: 1085–93.

18 Garvey WT, Batterham RL, Bhatta M, *et al.* Two-year effects of semaglutide in adults with overweight or obesity: the STEP 5 trial. *Nat Med* 2022; 28: 2083–91.

19 Lind M, Hirsch IB, Tuomilehto J, *et al.* Liraglutide in people treated for type 2 diabetes with multiple daily insulin injections: randomised clinical trial (MDI Liraglutide trial). *BMJ* 2015; : h5364.

20 Marre M, Shaw J, Brändle M, *et al.* Liraglutide, a once‐daily human GLP‐1 analogue, added to a sulphonylurea over 26 weeks produces greater improvements in glycaemic and weight control compared with adding rosiglitazone or placebo in subjects with Type 2 diabetes (LEAD‐1 SU). *Diabetic Medicine* 2009; 26: 268–78.

21 Dejgaard TF, Schmidt S, Frandsen CS, *et al.* Liraglutide reduces hyperglycaemia and body weight in overweight, dysregulated insulin‐pump‐treated patients with type 1 diabetes: The Lira Pump trial—a randomized, double‐blinded, placebo‐controlled trial. *Diabetes Obes Metab* 2020; 22: 492–500.

22 Bailey TS, Takács R, Tinahones FJ, *et al.* Efficacy and safety of switching from sitagliptin to liraglutide in subjects with type 2 diabetes ( <scp>LIRA‐SWITCH</scp> ): a randomized, double‐blind, double‐dummy, active‐controlled 26‐week trial. *Diabetes Obes Metab* 2016; 18: 1191–8.

23 de Wit HM, Vervoort GM, Jansen HJ, de Galan BE, Tack CJ. Durable efficacy of liraglutide in patients with type 2 diabetes and pronounced insulin‐associated weight gain: 52‐week results from the Effect of Liraglutide on insulin‐associated <scp>wE</scp> ight <scp>GA</scp> iN in patients with Type 2 diabetes’ ( <scp>ELEGANT</scp> ) randomized controlled trial. *J Intern Med* 2016; 279: 283–92.

24 Davies MJ, Bain SC, Atkin SL, *et al.* Efficacy and Safety of Liraglutide Versus Placebo as Add-on to Glucose-Lowering Therapy in Patients With Type 2 Diabetes and Moderate Renal Impairment (LIRA-RENAL): A Randomized Clinical Trial. *Diabetes Care* 2016; 39: 222–30.

25 Davies M, Færch L, Jeppesen OK, *et al.* Semaglutide 2·4 mg once a week in adults with overweight or obesity, and type 2 diabetes (STEP 2): a randomised, double-blind, double-dummy, placebo-controlled, phase 3 trial. *The Lancet* 2021; 397: 971–84.

26 Blonde L, Belousova L, Fainberg U, *et al.* Liraglutide as add‐on to sodium‐glucose co‐transporter‐2 inhibitors in patients with inadequately controlled type 2 diabetes: LIRA‐ADD2SGLT2i, a 26‐week, randomized, double‐blind, placebo‐controlled trial. *Diabetes Obes Metab* 2020; 22: 929–37.

27 Levin P, Wei W, Wang L, Pan C, Douglas D, Baser O. Combination therapy with insulin glargine and exenatide: real-world outcomes in patients with type 2 diabetes. *Curr Med Res Opin* 2012; 28: 439–46.

28 Barnett AH, Burger J, Johns D, *et al.* Tolerability and efficacy of exenatide and titrated insulin glargine in adult patients with type 2 diabetes previously uncontrolled with metformin or a sulfonylurea: A multinational, randomized, open-label, two-period, crossover noninferiority trial. *Clin Ther* 2007; 29: 2333–48.

29 Aroda VR, Rosenstock J, Terauchi Y, *et al.* PIONEER 1: Randomized Clinical Trial of the Efficacy and Safety of Oral Semaglutide Monotherapy in Comparison With Placebo in Patients With Type 2 Diabetes. *Diabetes Care* 2019; 42: 1724–32.

30 Aroda VR, Frias JP, Ji L, *et al.* Efficacy and safety of <scp>once‐weekly</scp> efpeglenatide in people with suboptimally controlled type 2 diabetes: The <scp>AMPLITUDE‐D</scp> , <scp>AMPLITUDE‐L</scp> and <scp>AMPLITUDE‐S</scp> randomized controlled trials. *Diabetes Obes Metab* 2023; 25: 2084–95.
